# Complete Genome Sequences of Three Novel *Saimiri sciureus* Papillomavirus Types Isolated from the Cervicovaginal Region of Squirrel Monkeys

**DOI:** 10.1128/genomeA.01400-17

**Published:** 2018-01-04

**Authors:** Zigui Chen, Charles E. Wood, Christian R. Abee, Robert D. Burk

**Affiliations:** a Department of Microbiology, Faculty of Medicine, The Chinese University of Hong Kong, Hong Kong SAR, China; b Department of Pathology, Wake Forest School of Medicine, Winston-Salem, North Carolina, USA; c University of Texas MD Anderson Cancer Center, Houston, Texas, USA; d Department of Pediatrics, Albert Einstein College of Medicine, Bronx, New York, USA; e Department of Microbiology and Immunology, Albert Einstein College of Medicine, Bronx, New York, USA; f Department of Epidemiology and Population Health, Albert Einstein College of Medicine, Bronx, New York, USA; g Department of Obstetrics & Gynecology and Women’s Health, Albert Einstein College of Medicine, Bronx, New York, USA

## Abstract

The complete genome sequences of three novel *Saimiri sciureus* papillomavirus (SscPV) types (SscPV1 to SscPV3) isolated from the cervicovaginal region of squirrel monkeys were characterized. These three PV types share 78.1 to 83.3% nucleotide sequence identities with each other across the complete L1 open reading frame and cluster in the genus *Dyoomikronpapillomavirus*.

## GENOME ANNOUNCEMENT

Papillomaviruses (PVs) are an ancient and heterogeneous family of circular double-stranded DNA viruses about 8 kb in size that infect the epithelial surfaces of skin, oral, and anogenital sites from a large spectrum of vertebrates, including reptiles, fishes, birds, mammals, and human beings. A distinct human PV (HPV) type is defined when the complete L1 open reading frame (ORF) is less than 90% similar to characterized types (1, 2). Currently, approximately 200 types of human papillomaviruses (HPVs) and 160 types of animal papillomaviruses have been characterized. Nonhuman primate (NHP) papillomaviruses have been detected from the cutaneous and mucosal sites of a wide range of apes and Old World monkeys, including rhesus (*Macaca mulatta*) and cynomolgus (*Macaca fascicularis*) macaques (3–5), baboon monkeys (*Papio hamadryas*) (6), colobine monkeys (*Colobus guereza*) (7, 8), and common (*Pan troglodytes*) and pygmy (*Pan paniscus*) chimpanzees (9). A large set of *Macaca fascicularis* papillomaviruses (MfPVs) (e.g., those within the species *Alphapapillomavirus* 12) have been isolated from the cervicovaginal region of macaques, some of which are associated with macaque cervical cancer (5, 10).

In this report, we characterize the complete genome sequences of three novel papillomavirus types isolated from the cervicovaginal region of squirrel monkeys (*Saimiri sciureus*). The viral DNA was purified from exfoliated cervical cells and screened using PCR-based MY09/11 and FAP59/64 primer systems (11, 12). Sequences from the PCR products were compared with a PV database maintained by the Burk lab using a BLASTN search and shown to have <90% similarities to the characterized PV types. The whole genomes were PCR amplified as two overlapping fragments using degenerated primer sets designed on the available L1 gene sequences and the consensus E1 alignments. PCR products of appropriate size were cloned into the TOPO TA pCR2.1 vector (Invitrogen, Carlsbad, CA) and subsequently Sanger sequenced using primer walking at the Einstein Sequencing Facility, New York. Geneious R9.1.7 (13) was used to assemble segmented sequences to the complete genome sequences and identify ORFs.

These three novel genomes were named *Saimiri sciureus* papillomavirus types 1, 2, and 3 (SscPV1 to SscPV3), with sizes of 7,596 bp, 7,657 bp, and 7,664 bp, respectively. All three genomes contain five putative early genes (E6, E7, E1, E2, and E4) and two late genes (L2 and L1). The complete L1 ORFs of these three types share 78.1 to 83.3% nucleotide sequence pairwise identities with each other and differ by >26% from any other known PVs, indicating that these isolates meet the criteria to define novel types. A phylogenetic analysis using either L1 ORFs or complete genomes indicates that they cluster into a monophyletic clade within the genus *Dyoomikronpapillomavirus* and represent the species *Dyoomikronpapillomavirus 1*, together with *Alouatta guariba* papillomavirus 1 (AgPV1) (GenBank accession no. KP861980). The characterization of these novel primate PVs expands the range of hosts to include New World monkeys and extends the ancient isolates of PVs closely related to the alphapapillomaviruses. Further investigations on the epidemiology, evolution, and pathogenesis of *Saimiri sciureus* papillomaviruses are warranted.

### Accession number(s).

The complete genome sequences of SscPV1 to SscPV3 were submitted to the NCBI/GenBank database with accession numbers JF304765 to JF304767.
